# Influencing electroclinical features and prognostic factors in patients with anti-NMDAR encephalitis: a cohort follow-up study in Chinese patients

**DOI:** 10.1038/s41598-020-67485-6

**Published:** 2020-07-01

**Authors:** Yingxin Wang, Ailiang Miao, Yongwei Shi, Jianqing Ge, Lingling Wang, Chuanyong Yu, Haiyan Xu, Yuanwen Yu, Shuyang Huang, Yihan Li, Xiaoshan Wang

**Affiliations:** 1 Department of Neurology, The Affiliated Brain Hospital of Nanjing Medical University, Nanjing Medical University, Guang Zhou Road 264, Nanjing, 210029 Jiangsu China; 2 Department of Video-Electroencephalogram, The Affiliated Brain Hospital of Nanjing Medical University, Nanjing Medical University, Nanjing, Jiangsu China; 3 Department of Neurology, Taizhou Fourth People’s Hospital, Taizhou, Jiangsu China; 4 Medical Records Room, Nanjing Brain Hospital, Nanjing Medical University, Nanjing, Jiangsu China

**Keywords:** Immunology, Neuroscience, Neurology

## Abstract

The clinical manifestations of patients with anti-*N*-methyl-d-aspartate receptor (anti-NMDAR) encephalitis in East China and factors associated with prognosis were analyzed. A retrospective study of 106 patients (58 females; 48 males) with anti-NMDAR encephalitis in East China was carried out from June 2015 to February 2019. Clinical features and factors influencing outcomes were reviewed. Behavioral changes were observed in 74.5% (79/106) of patients, and comprised the initial symptoms in 61.3% (65/106). Seizures were observed in 67% (71/106) of patients, and served as initial symptoms in 31.1% (33/106). A total of 54.9% (39/71) of seizures were focal seizures. More clinical symptoms were observed in female patients than in male patients (*P* = 0.000). Similarly, background activity (BA) with high cerebrospinal fluid (CSF) antibody titers at the peak stage was more severe in female patients than in male patients (*P* = 0.000). The Binary logistic regression and receiver operating characteristic (ROC) curve analyses revealed the factors associated with poor outcomes included consciousness disturbance (OR 4.907, 95% CI 1.653–14.562, *P* = 0.004; area: 65.4%, sensitivity: 44.2%, specificity: 86.5%, *P* = 0.014), EEG BA (OR 3.743, 95% CI 1.766–7.932, *P* = 0.001; area: 76.6%, sensitivity: 73%, specificity: 75%, *P* = 0.000), number of symptoms (OR 2.911, 95% CI 1.811–4.679, *P* = 0.000; area: 77.1%, sensitivity: 59.5%, specificity: 78.6%, *P* = 0.000) and CSF antibody titer (OR 31.778, 95% CI 8.891–113.57, *P* = 0.000; area: 83.9%, sensitivity: 89.2%, specificity: 78.6%, *P* = 0.000). EEG BA and number of symptoms were associated with CSF antibody titers. Consciousness disturbances, EEG BA, number of symptoms and CSF antibody titers served as predictors of poor outcomes.

## Introduction

Anti-*N*-methyl-d-aspartate receptor (anti-NMDAR) encephalitis is an autoimmune disease associated with IgG antibodies against the NR1 subunit of the NMDA receptor^1,2^. Patients develop subacute psychiatric symptoms, memory loss, movement disorders and seizures. Seizures can occur at any stage but most commonly manifest early^3^. Magnetic resonance imaging (MRI) remains one of the most important examinations for the diagnosis of central nervous system diseases. As reported, normal brain MRI results have been observed in most patients^3–5^. Electroencephalography (EEG) may be useful for diagnosing anti-NMDAR encephalitis^6–8^. The number of clinical symptoms and electroencephalography (EEG) results were associated with cerebrospinal fluid (CSF) antibody titers^9^. To date, no study has established whether EEG background activity (BA) during the peak stage of anti-NMDAR encephalitis is associated with prognosis. Currently available data are also unclear regarding the associations between CSF antibody titers and outcomes.

The purpose of our study is to provide a summary of the clinical features, and analyze the association between clinical features and prognosis in patients with anti-NMDAR encephalitis.

## Patients and methods

### Patients

A total of 106 patients were included from the Department of Neurology of Nanjing Brain Hospital. The anti-NMDAR encephalitis diagnostic criteria were as follows: (1) patients with positive anti-NMDAR IgG in CSF with or without positive anti-NMDAR IgG in serum; and (2) patients with subacute development (disease course < 3 months) of one or more symptoms, including psychiatric symptoms, cognitive impairment, seizures, language impairment, consciousness disturbance, involuntary movement, autonomic nervous system dysfunction, or central hypoventilation. If patients are diagnosed with other diseases, such as brain tumor, viral encephalitis, metabolic diseases, and drug poisoning, they were excluded^10^.

### Patient data

The following patient data were collected: age; gender; malignancy (teratomas and others); neurological symptoms such as psychiatric symptoms and seizures; anti-NMDAR titers; EEG BA; extreme delta brush (EDB); arterial spin labeling (ASL); MRI; lumbar puncture; and modified Rankin scale (mRS) scores after 12 to 50 months of immunosuppressive treatment^8^.

According to the previous study^11^ and our clinical observation, 1:1 and 1:3.2 CSF anti-NMDAR titers was defined as low antibody titers; 1:10 and 1:32 CSF anti-NMDAR titers were defined as high antibody titers.

According to the previous study^6^ and EEG works^12–14^, the EEG BA was classified the normal, mild diffuse polymorphic slowing (mild DPS), moderate diffuse polymorphic slowing (moderate DPS), and severe diffuse polymorphic slowing (SDPS). BA: Any underlying EEG activity representing the setting in which focal or transient activity, either normal or abnormal, appears and from which such underlying pattern is distinguished^12^. Diffuse: Occurring over large areas of the head^12^. Normal adult EEG^13^: The occipital leads show rhythm in the alpha range (9–13 Hz). Frontal and central leads show faster activity. The posterior dominant alpha is present in relaxed wakefulness and attenuates with eye opening and disappears as the patient falls into drowsiness and sleep. Mild DPS^14^: 8 Hz posterior rhythm; diffuse theta increases (≥ 10% of awake records in normal subjects). Moderate DPS^14^: 7 to 8 Hz posterior rhythm; diffuse arrhythmic ≤ 4 Hz waves increase. SDPS^14^: Delta and theta replaces alpha as highest-frequency background rhythm; arrhythmic and/or rhythmic ≤ 4 Hz diffuse waves increase. EDB^6^: Delta activity with superimposed bursts of rhythmic 20–30 Hz beta frequency activity “riding” on each delta wave.

The initial stage: 14 days after symptom onset; the peak stage: 14 to 60 days after symptom emergence; the improvement stage: 60 to 180 days after disease onset; and the recovery stage: 180 days after disease onset^15,16^.

### Statistics

The Statistical Package for Social Sciences was used to performed statistical analyses (IBM Corporation, Armonk, NY, USA). The Mann–Whitney U test was performed to compare EEG categorical variables at the peak stage between patients with low and high CSF antibody titers and between female and male patients. The independent-samples *t* test was adopted to compare symptoms between patients with low and high CSF antibody titers, and between female and male patients, as well as to compare CSF white cell and protein levels between patients with abnormal T2/fluid-attenuated inversion recovery (FLAIR) image and patients with normal T2/FLAIR image. The binary logistic regression analysis and receiver operating characteristic (ROC) curve were used to analyze the association between age, sex, consciousness disturbance, CSF antibody titers, EEG BA during peak stage, number of symptoms, imaging results, relapse, intensive care unit (ICU) admissions, follow-up period and prognosis in patients with anti-NMDAR encephalitis. P < 0.05 indicated statistical significance. All methods in this study were carried out in accordance with relevant guidelines and regulations.

## Results

### Patient characteristics

**Figure 1:**
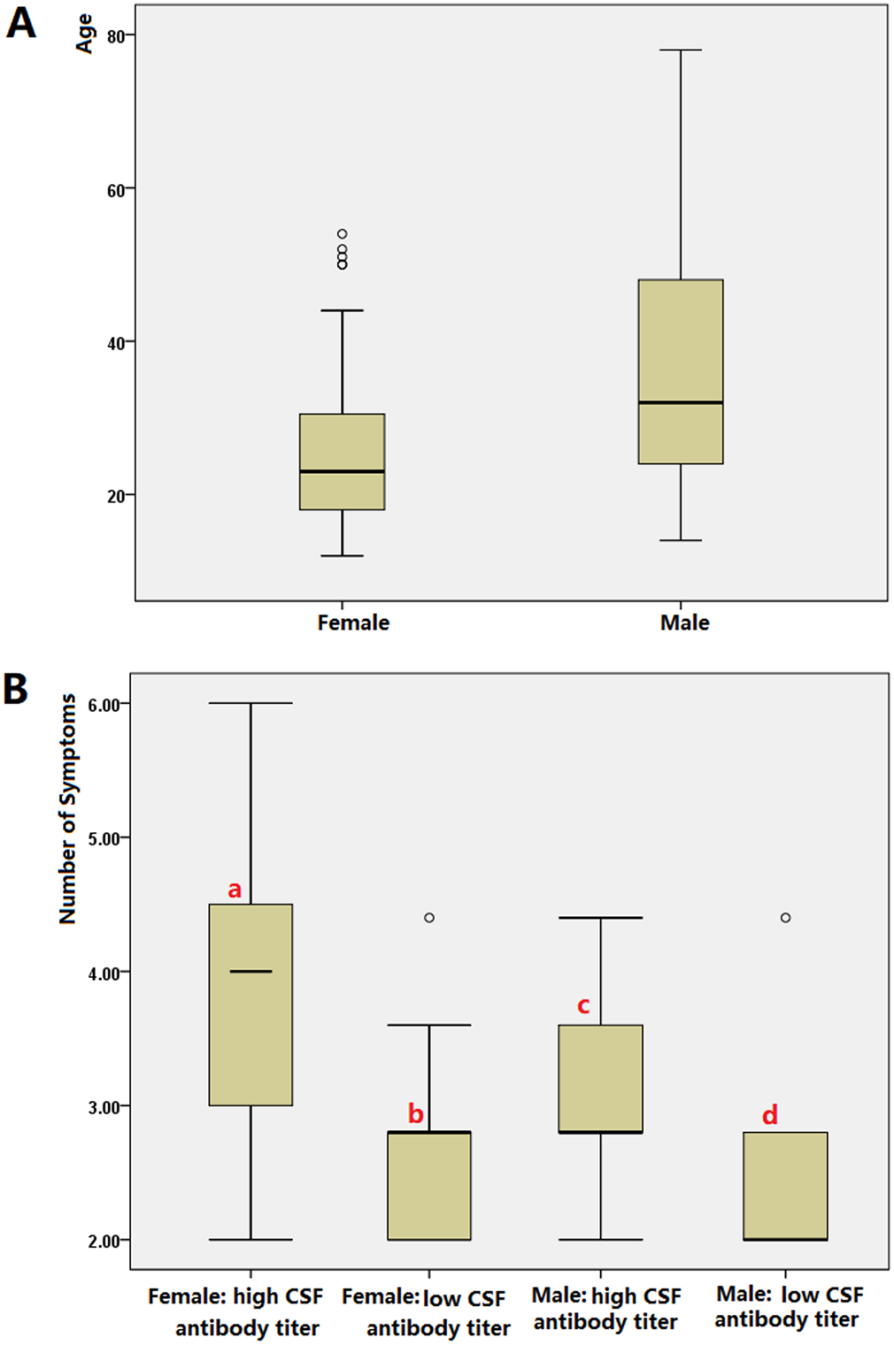
(**A**) The male patients were older than the female patients (36.49 ± 2.3 *vs* 25.94 ± 1.64, *P* = 0.000). (**B**) Patients with high CSF antibody titers experienced more clinical symptoms than those with low CSF antibody titers (a *vs* b: mean 4.06 ± 0.98 *vs* 1.88 ± 0.86, *P* = 0.000; c *vs* d: mean 2.29 ± 1.0 *vs* 1.50 ± 0.79, *P* = 0.006). Similarly, more clinical symptoms were observed in the female patients than in the male patients (a *vs* c: mean 4.06 ± 0.98 *vs* 2.29 ± 1.0, *P* = 0.000).

We retrospectively identified 106 patients with anti-NMDAR encephalitis (58 females; 48 males). The ages of the male patients were higher than those of the female patients (36.49 ± 2.3 *vs* 25.94 ± 1.64, *P* = 0.000; Fig. 1A, Table 1).

**Table 1:** Clinical Characteristics of patients with anti-NMDAR encephalitis.

Clinical features	Patients	Mean Std. deviation	*P* value
**Sex**			
Female	58		
Male	48		
**Age**			
Female_age		25.94 ± 1.64	
Male_age		36.49 ± 2.3	
Female_age *vs* male_age			*P* = 0.000^d^
^a^	Female 36; male 24		
^b^	Female 18; male 19		
**Symptom presentation**			
Behavioral changes			
Female	49 (42 OS)		
^a^	35 (29 OS)		
^b^	10 (9 OS)		
Male	30 (23 OS)		
^a^	15 (12 OS)		
^b^	11 (8 OS)		
**Seizures**			
Female	40 (14 OS; 22 FS)		
^a^	25 (4 OS; 12 FS)		
^b^	11 (6 OS; 8 FS)		
Male	31 (19 OS; 17 FS)		
^a^	15 (8 OS; 9 FS)		
^b^	11 (8 OS; 7 FS)		
**Consciousness disturbance**	32		
**Cognitive impairment**	59		
**Language impairment**	16		
**Focal limb weakness**	3		
**Involuntary movement**	16		
**Oral**	10 (female^a^)		
**Other**	6		
**Number of symptoms**			
Female^c^		4.06 ± 0.98 *vs* 1.88 ± 0.86	*P* = 0.000^d^
Male^c^		2.29 ± 1.0 *vs* 1.50 ± 0.79	*P* = 0.006^d^
Female *vs* male^a^		4.06 ± 0.98 *vs* 2.29 ± 1.0	*P* = 0.000^d^
Female *vs* male^b^		1.88 ± 0.86 *vs* 1.50 ± 0.79	*P* = 0.179^d^
**Imaging**			
Normal T2/FLAIR	74		
Abnormal T2/FLAIR	28		
Total ASL	23		
Abnormal T2/Flair and focal high blood flow	9		
Normal T2/Flair and focal high blood flow	13		
EEG during peak stage		Median 19.5	
**Background activity (BA)**			
Normal	2 female; 2 male		
Mild DPS	8 female; 17 male		
Moderate DPS	19 female; 12 male		
SDPS	13 female; 2 male		
EDB	5 female^a^; 1 male		
Female^c^			*P* = 0.000^e^
Male^c^			*P* = 0.461^e^
Female *vs* male^a^			*P* = 0.000^e^
Female *vs* male^b^			*P* = 0.657^e^
**CSF**			
White cells			
Abnormal T2/FLAIR		89.63 ± 29.53	
Normal T2/FLAIR		18.44 ± 7.42	*P* = 0.000^d^
Abnormal T2/FLAIR *vs* normal			
T2/FlAIR			
Normal T2/FLAIR and focal high blood flow		4.9 ± 8.52	
Protein			
Abnormal T2/Flair		0.63 ± 0.06	*P* = 0.002^d^
Normal T2/Flair		0.4 ± 0.03	
Abnormal T2/Flair *vs* normal T2/Flair			
ICU admission	9^a^; 2^b^		
**Tumor**			
Ovarian teratomas	4		
Other	1		
**Therapy**			
First-line alone	103		
First-line and second-line	3		
**With/without relapse**			
Female	3^a^; 1^b^		
Male	4^a^		
**mRS (follow-up from 12 to 50 months)**	Median 19		
0–1	37		
≥ 2	53 (4 died)		

*Std. deviation* standard deviation, *mild DPS* mild diffuse polymorphic slowing, *moderate DPS* moderate diffuse polymorphic slowing, *SDPS* severe diffuse polymorphic slowing, *EDB* extreme delta brush, *ASL* arterial spin labeling, *CSF* cerebrospinal fluid.

^a^High CSF antibody titer (1:10 or 1:32).

^b^Low CSF antibody titer (1:1 or 1:3.2).

^c^Low CSF antibody titer (1:1 or 1:3.2) *vs* high CSF antibody titer (1:10 or 1:32).

^d^Independent-sample *t* test.

^e^Mann–Whitney *U* test. Onset denotes the number of patients with the clinical symptoms manifesting onset symptoms.

### Clinical characteristics

High CSF antibody titers were observed in 60 patients with anti-NMDAR encephalitis and low CSF antibody titers in 37 patients. The twice positive anti-NMDAR IgG in serum were observed in the remaining 9 patients with unsuccessful lumber puncture. The forty female patients with anti-NMDAR encephalitis were performed the gynecological sonography, pelvic MRI or CT. Ovarian teratomas were observed in 6 female patients, and 1 patient manifested pituitary microadenoma. Eight patients experienced relapse. Eleven patients were admitted the ICU.

Behavioral changes were observed in 74.5% (79/106) of the patients. Behavioral changes comprised the initial symptoms in 61.3% (65/106). Seizures were observed in 67% (71/106) of the patients, and 54.9% (39/71) of seizures were focal seizures. Seizures served as initial symptoms in 31.1% (33/106) of the patients. Constant chewing was noted in 10 female patients with high CSF antibody titers during the peak clinical state. The patients with high CSF antibody titers experienced more clinical symptoms than those with low CSF antibody titers (female mean: 4.06 ± 0.98 *vs* 1.88 ± 0.86, respectively, *P* = 0.000; male mean: 2.29 ± 1.0 *vs* 1.50 ± 0.79, *P* = 0.006). Similarly, more clinical symptoms were observed in the female patients than in the male patients (mean: 4.06 ± 0.98 *vs* 2.29 ± 1.0, *P* = 0.000) (Fig. 1B, Table 1).

**Figure 2:**
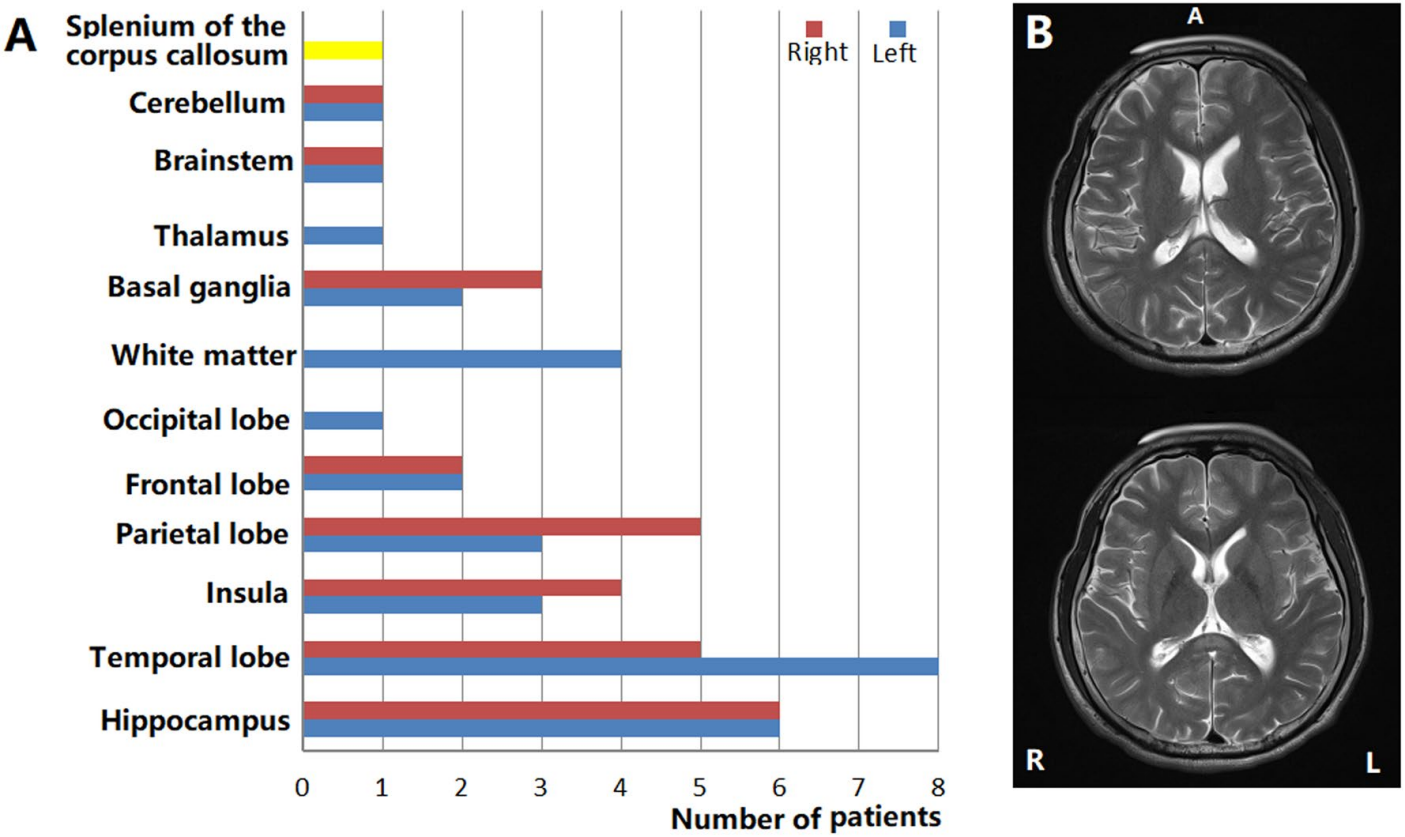
(**A**) Brain lesions in patients with anti-NMDAR encephalitis. (**B**) Splenium of the corpus callosum lesions in patient with anti-NMDAR encephalitis.

**Figure 3:**
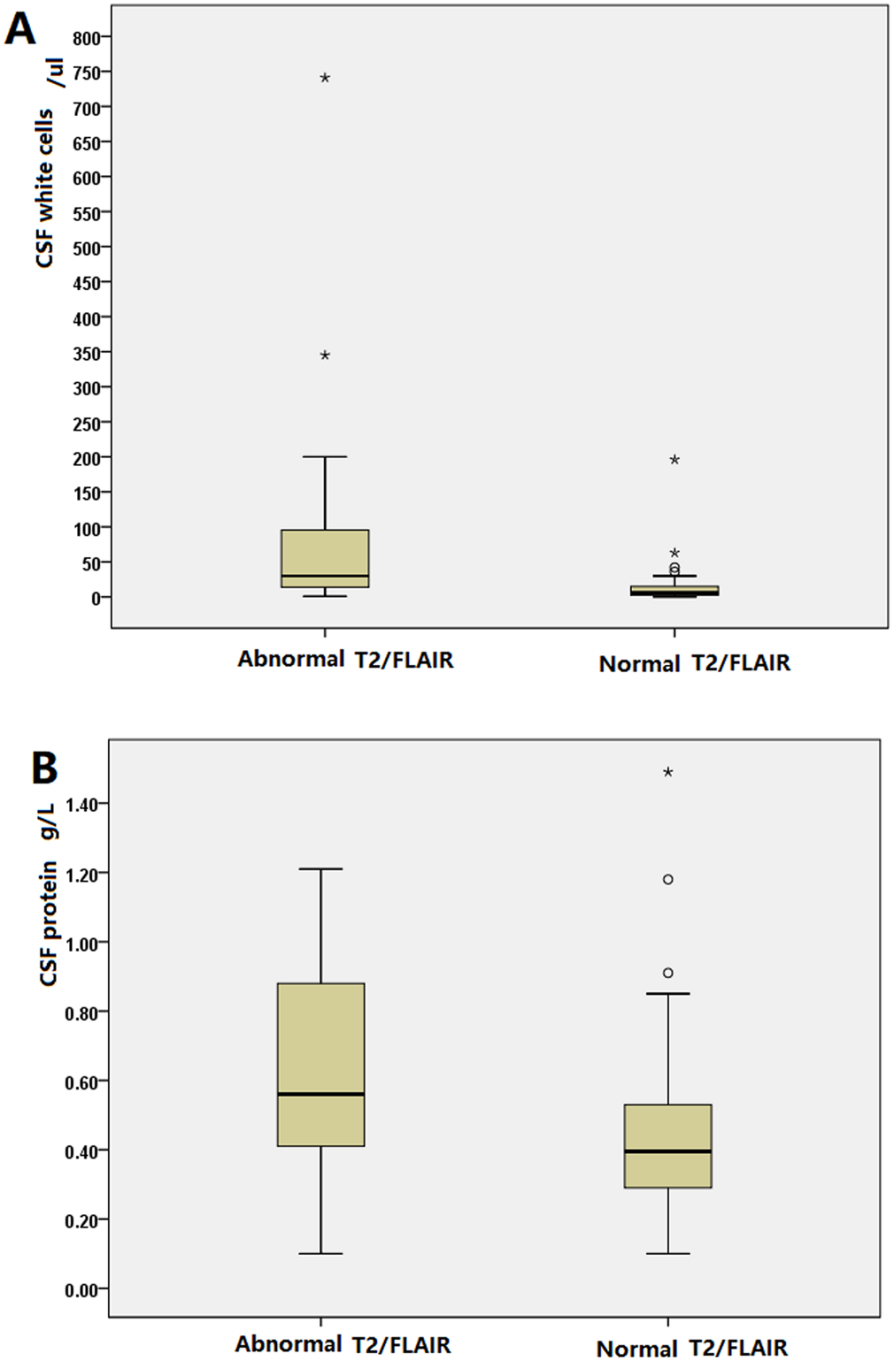
(**A**) CSF white cells are higher in patients with abnormal T2/FLAIR than in patients with normal T2/FLAIR (mean: 89.63 ± 29.53 *vs* 18.44 ± 7.42, *P* = 0.000). (**B**) CSF protein levels are higher in patients with abnormal T2/FLAIR than in patients with normal T2/FLAIR (mean: 0.63 ± 0.06 *vs* 0.4 ± 0.03, *P* = 0.002).

A total of 72.5% (74/102) of patients showed normal MRIs. Brain lesions were observed in 27.45% of patients (28/102). On MRI, hyperintensities involving the hippocampus, temporal cortex, insula, parietal cortex, frontal cortex, occipital lobe, white matter, basal ganglia, thalamus, brainstem, cerebellum, and splenium of the corpus callosum were noted in 11.8% (12/102), 12.7% (13/102), 6.9% (7/102), 7.8% (8/102), 3.9% (4/102), 0.98% (1/102), 3.9% (4/102), 2.9% (3/102), 0.98% (1/102), 0.98% (1/102), 0.98% (1/102), and 0.98% (1/19) of the patients, respectively (Table 1, Fig. 2). During the peak stage of the disease, focal high blood flow with normal MRIs were observed from 56.52% (13/23) ASL of anti-NMDAR encephalitis patients in Table 1. Focal high blood flow with brain lesions were observed in 39.13% (9/23) ASL of patients (Table 1). CSF white cells in patients with abnormal T2/FLAIR were higher than those in patients with normal T2/FLAIR (mean: 89.63 ± 29.53 *vs* 18.44 ± 7.42, respectively, *P* = 0.000). Similarly, CSF protein levels were higher in patients with abnormal T2/FLAIR than in patients with normal T2/FLAIR (mean: 0.63 ± 0.06 *vs* 0.4 ± 0.03, *P* = 0.002) (Table 1, Fig. 3).

**Figure 4:**
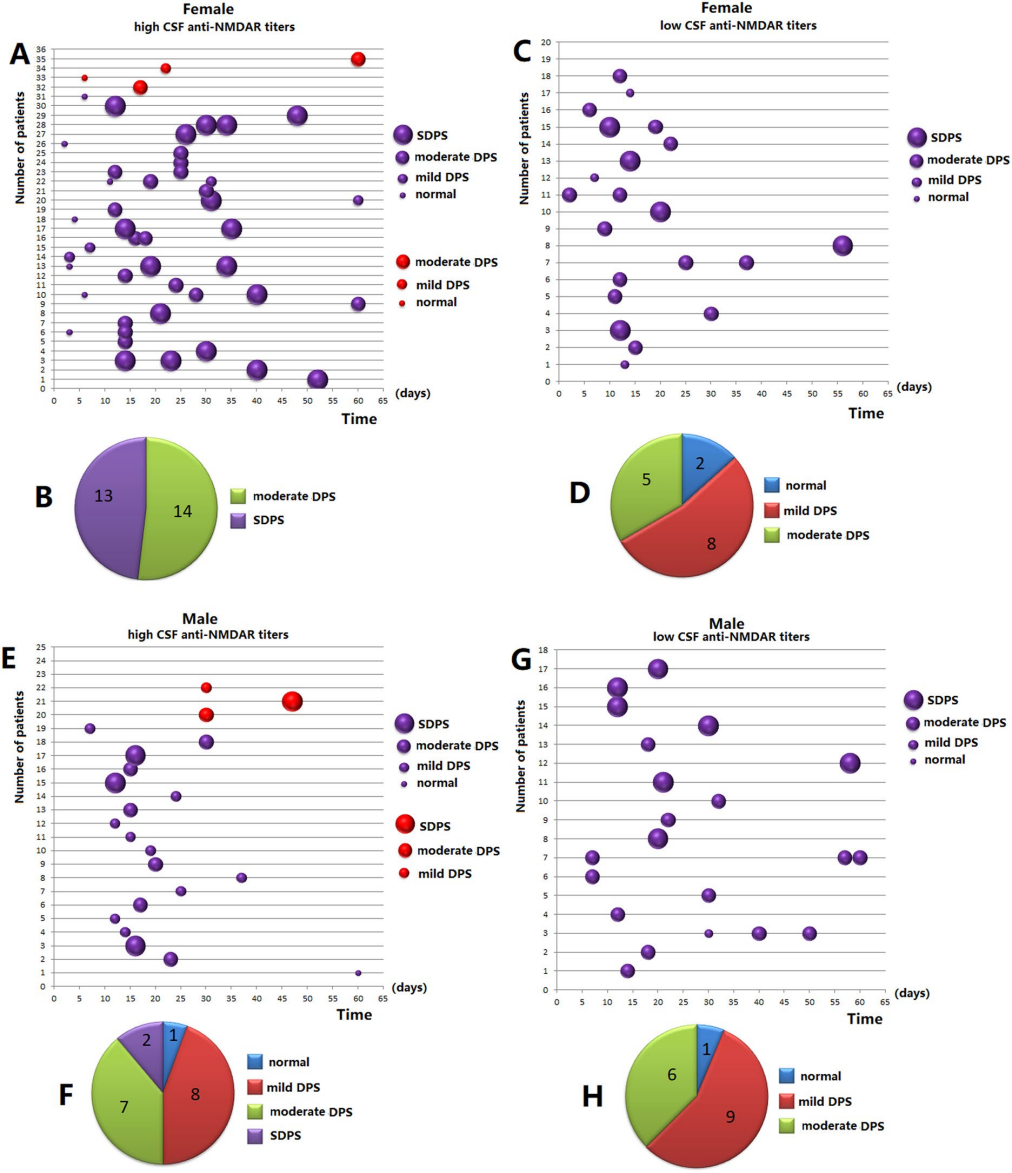
One hundred and fourteen EEG or VEEG recordings were obtained from 92 patients. EEG BA was significantly aggravated at 12 days (**A**,**C**,**E**,**G**). A total of 107 EEGs and CSF antibody titers were collected from 85 patients. 76 first EEGs ranging from 12 to 60 days (median 19.5 days) from 76 patients were analyzed. SDPS was observed in 15 anti-NMDAR encephalitis patients; moderate DPS in 32 patients; mild DPS in 25 patients, and normal in 4 patients (Table 1). (**B** *vs* **D**) BA during the peak stage in female patients with high CSF antibody titers was more severe than that in patients with low CSF antibody titers (Mann–Whitney *U* test, *P* = 0.000). (**B** *vs* **F**) BA with high CSF antibody titers at the peak stage was more severe in female patients than in male patients (Mann–Whitney *U* test, *P* = 0.000). (**H**) SDPS was not observed in male patients with low CSF antibody titers. *Mild DPS* mild diffuse polymorphic slowing, *Moderate DPS* moderate diffuse polymorphic slowing, *SDPS* severe diffuse polymorphic slowing. Purple bubble patients with EEGs and CSF antibody titers. Red bubble with EEGs and unclear CSF antibody titers: X-axis: time. Y-axis: number of patients.

One hundred and fourteen EEG or VEEG recordings were obtained from 92 patients. EEG BA was significantly aggravated at 12 days (Fig. 4 A,C,E,G). A total of 107 EEGs and CSF antibody titers were collected from 85 patients. 91 EEGs and CSF antibody titers during 12 to 60 days from 76 patients were obtained. 76 first EEGs during from 12 to 60 days (median 19.5 days) from 76 patients were analyzed. SDPS was observed in 15 anti-NMDAR encephalitis patients, moderate DPS in 32 patients, mild DPS in 25 patients, and normal activity in 4 patients, separately (Table 1, Fig. 4B,D,F,H). The BA during the peak stage in female patients with high CSF antibody titers was more severe than that in female patients with low CSF antibody titers (Fig. 4B *vs* C; Mann–Whitney *U* test, *P* = 0.000). BA in those with high CSF antibody titers at the peak stage was more severe in female patients than in male patients (Fig. 4B *vs* F; Mann–Whitney *U* test, *P* = 0.000) (Table 1, Fig. 4).

### Factor-related prognosis

A total of 103 (97.2%) patients were treated with first-line immunotherapy, 3 (2.8%) with second-line immunotherapy, and 1 with tumor removal and immunotherapy.

**Figure 5:**
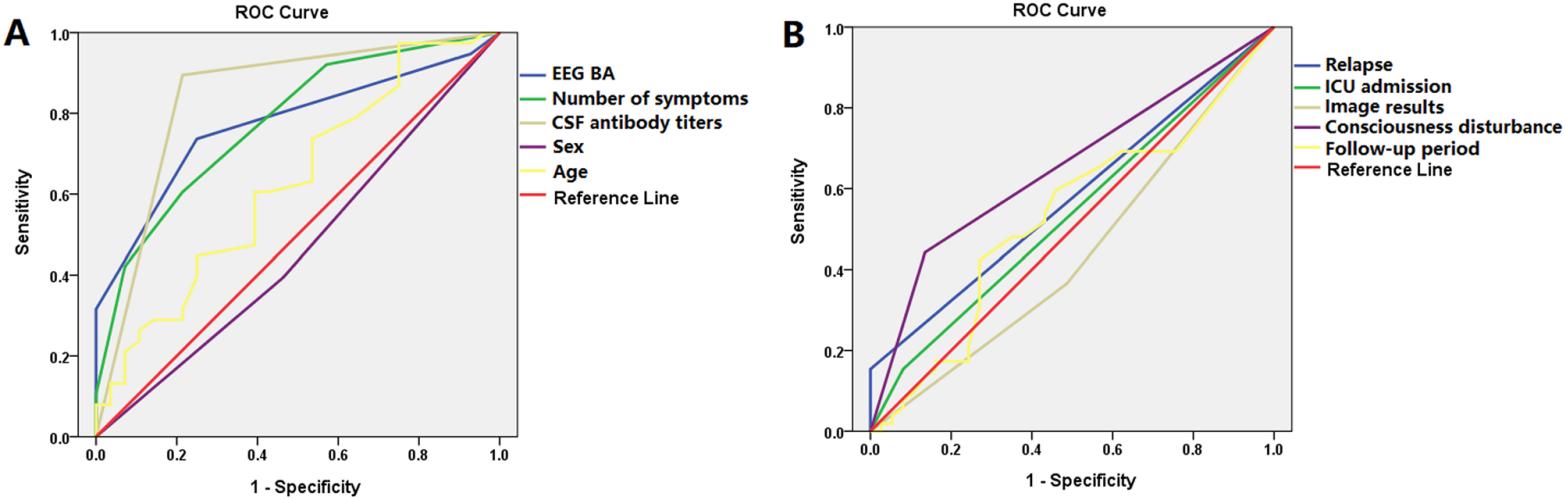
(**A**) The predictors for poor outcomes included EEG BA (area: 76.6%, sensitivity: 73%, specificity: 75%, *P* = 0.000), number of symptoms (area: 77.1%, sensitivity: 59.5%, specificity: 78.6%, *P* = 0.000) and CSF antibody titers (area: 83.9%, sensitivity: 89.2%, specificity: 78.6%, *P* = 0.000) in the ROC curve analysis. (**B**) The consciousness disturbance was related to poor outcomes (area: 65.4%, sensitivity: 44.2%, specificity: 86.5%, *P* = 0.014, (**B)**), *BA* background activity.

At follow-up after at least 12 months (median: 19 months; range: 12 to 50 months), 37 patients reached a mRS of 0–2, and 53 patients had poor outcomes (mRS ≥ 2) (Table 1). The follow-up period for 16 patients was < 12 months. The age, sex, EEG BA, number of symptoms, CSF antibody titers, consciousness disturbance, imaging results, relapse, ICU admission, follow-up period, and prognosis were analyzed with binary logistic regression analysis and ROC curve analysis. In the binary logistic regression analysis, the factors associated with poor outcomes included consciousness disturbance (OR 4.907, 95% CI 1.653–14.562, *P* = 0.004), EEG BA (OR 3.743, 95% CI 1.766–7.932, *P* = 0.001), number of symptoms (OR 2.911, 95% CI 1.811–4.679, *P* = 0.000) and CSF antibody titers (OR 31.778, 95% CI 8.891–113.57, *P* = 0.000) (Table 2, Fig. 5). In the ROC curve analysis, predictors for poor outcomes included consciousness disturbance (area: 65.4%, sensitivity: 44.2%, specificity: 86.5%, *P* = 0.014), EEG BA (area: 76.6%, sensitivity: 73%, specificity: 75%, *P* = 0.000), number of symptoms (area: 77.1%, sensitivity: 59.5%, specificity: 78.6%, *P* = 0.000) and CSF antibody titers (area: 83.9%, sensitivity: 89.2%, specificity: 78.6%, *P* = 0.000) (Table 2, Fig. 5).

**Table 2:** Factors associated with prognosis in patients with anti-NMDAR encephalitis.

Variables	PRC	*P* values	OR values	95% CI	
				Lower	Upper
**Binary logistic regression analysis**					
Consciousness disturbance	1.591	0.004	4.907	1.653	14.562
EEG BA	1.32	0.001	3.743	1.766	7.932
Number of symptoms	1.068	0.000	2.911	1.811	4.679
CSF antibody titers	3.459	0.000	31.778	8.891	113.57

Variables	Area (%)	*P* values	Sensitivity (%)	Specificity (%)
**ROC curve**				
Consciousness disturbance	65.4%	0.014	44.2	86.5
EEG BA	76.6%	0.000	73	75
Number of symptoms	77.1%	0.000	59.5	78.6
CSF antibody titers	83.9%	0.000	89.2	78.6

*EEG BA* EEG background activity during peak stage, *PRC* partial regression coefficient.

## Discussion

Our study focused on clinical features and factors related to prognosis in patients with anti-NMDAR encephalitis. The male patients were older than the female patients (*P* = 0.000) (Table 1). There was no sex difference in this sample, which is similar to a previous report on adult-onset anti-NMDAR encephalitis in Korea^17^. The prevalence of teratomas in females over 18 years old was 56%, but only 31% in females under 18 years old, and only 9% in females under 14 years old^3^. There were only 15% female patients with ovarian teratoma in this study. In addition to age and sex, the presence of an ovarian teratoma is also dependent on ethnicity^3^.

Behavioral changes and seizures were the major symptoms. Behavioral changes comprised the initial symptoms in 61.3% (65/106) of the patients with anti-NMDAR encephalitis in this study. Seizures were observed as the onset symptoms in 31.3% (33/106) of the patients. Moreover, 54.9% (39/71) of the seizures were focal (Table 1). Behavioral changes and seizures served as initial symptoms in most patients, which aligned with previous findings^18,19^. In an observational cohort study, 87% of patients exhibited 4 or more categories of symptoms by the end of the first month^20^. This study investigated whether the number of clinical symptoms related with CSF antibody titers and gender in patients with anti-NMDAR encephalitis. A greater number of clinical symptoms were observed in patients with high CSF antibody titers than in those with low CSF antibody titers (Table 1, Fig. 1), which aligned with our previous findings^9^. The BA during the peak-stage was worse in the female patients with high CSF antibody titers than in those with low CSF antibody titers (*P* = 0.000) (Table 1, Fig. 4). The number of clinical symptoms were greater in the female patients with high CSF antibody titers than in male patients with high CSF antibody titers (mean: 3.97 ± 0.17 *vs* 2.33 ± 0.20, respectively, *P* = 0.000) (Table 1, Fig. 1). Meanwhile, the peak stage BA was worse in the female patients with high CSF antibody titers than in the male patients with high CSF antibody titers (*P* = 0.000) (Table 1, Fig. 4). The constant chewing was observed in the female patients with high CSF antibody titers during the peak stage, which may be a useful marker of the peak period of the disease. During the constant chewing, no epileptiform discharges (EDs) were observed in the EEG recordings, and antiepileptic drugs (AEDs) were ineffective^9^.

In several previous studies, 37.2–50% of patients showed abnormal brain MR imaging results^3–5^. During the peak stage of the disease, brain lesions were observed in 27.45% of the patients (28/102) in this study. On MRI, hyperintensities involving the hippocampus, temporal cortex, insula, parietal cortex, frontal cortex, and white matter (frontal region) were noted in 11.8% (12/102), 12.7% (13/102), 6.9% (7/102), 7.8% (8/102), 3.9% (4/102), and 3.9% (4/102) of the patients in this study, respectively (Table 1, Fig. 2). Moreover, 13 patients with normal MRIs showed focal high blood flow in Table 1. In a previous study, patients with abnormal MRIs were older than patients with normal MRIs^21^. There were no statistically significant differences in patients suffering seizures, MRI findings (abnormal or normal) between patients without or with tumors, suffering loss of consciousness, or patients suffering hypoventilation patients^21^. The prognosis evaluated by mRS was not affected by abnormal MRIs^21^. In this study, CSF white cells in the patients with abnormal T2/FLAIR were higher than those in the patients with normal T2/FLAIR (mean: 89.63 ± 29.53 *vs* 18.44 ± 7.42, respectively, *P* = 0.000). Similarly, there were higher CSF protein levels in patients with abnormal T2/FLAIR than in patients with normal T2/FLAIR (mean: 0.63 ± 0.06 *vs* 0.4 ± 0.03, respectively, *P* = 0.002).

In a large unselected cohort of adults, a normal posterior rhythm in the first EEG recording at a median of 19 days from disease onset predicted a favorable clinical outcome, while a severely abnormal EEG was associated with a poor outcome^22^. Ordinal logistic regression showed that the presence of a normal posterior rhythm was associated with lower mRS at final follow-up^22^. Binary logistic regression analysis and ROC curve analysis showed that EEG BA during the peak clinical state (median: 19.5 days) was associated with poor outcomes in this study. The number of symptoms and CSF antibody were also predictors of poor outcomes (Table 2, Fig. 5). A greater number of clinical symptoms were noted in patients with high CSF antibody titers than in those with low CSF antibody titers (Table 1, Fig. 1). The main epitope targeted by the antibodies is in the extracellular N-terminal domain of the NR1 subunit. Patients’ antibodies decreased the numbers of cell-surface NMDA receptors and NMDA-receptor clusters in postsynaptic dendrites, an effect that could be reversed by antibody removal. The severity of anti-NMDAR encephalitis was associated with antibody titers^5^. Furthermore, consciousness disturbance was a factor associated with nonfavorable outcomes in this study (Table 2, Fig. 5), which was similar to a previous study^23^. In previous studies, the predictors of good outcomes were early treatment and lack of ICU admission^4^. In our study, 11 patients were admitted to the ICU. ICU stay was not a predictor of poor outcomes. Many patients are not admitted to the ICU due to the shortage of ICU beds. All patients admitted to the ICU had poor outcomes (mRS ≥ 2). Relapses were defined as the new onset of symptoms, or worsening of symptoms after at least 2 months of improvement or stabilization. Although relapses were not a predictor of poor outcomes in our study, all patients with relapse had poor outcomes (mRS ≥ 2). The follow-up period may have been short in our study. Titulaer et al. followed their anti-NMDAR encephalitis patients for a median duration of 24 months and 7.8% of patients in that study experienced one or more clinical relapses^4^.

## Conclusion

EEG BA and the number of symptoms were associated with CSF antibody titers. CSF white cell and protein levels in patients with abnormal T2/FLAIR were higher than in patients with normal T2/FLAIR. Consciousness disturbance, EEG BA, number of symptoms and CSF antibody titers served as predictors of poor outcomes.

### Ethics approval and consent to participate

Our study was reviewed and approved by the ethical boards of the Affiliated Brain Hospital of Nanjing Medical University, and written informed consent was obtained from the family of all participants.
